# Blood Biomarkers Predict 10-Year Clinical Outcomes in Adult Patients With Congenital Heart Disease

**DOI:** 10.1016/j.jacadv.2024.101130

**Published:** 2024-07-27

**Authors:** Paul M. Hendriks, Annemien E. van den Bosch, Laurie W. Geenen, Vivan J.M. Baggen, Jannet A. Eindhoven, Robert M. Kauling, Judith A.A.E. Cuypers, Eric Boersma, Jolien W. Roos-Hesselink

**Affiliations:** a Department of Cardiology, Erasmus MC, University Medical Centre Rotterdam, Rotterdam, The Netherlands; b ERN-GUARD-Heart: European Reference Network for Rare and Low Prevalence Complex Diseases of the Heart; c Department of Clinical epidemiology, Erasmus MC, University Medical Centre Rotterdam, Rotterdam, The Netherlands

**Keywords:** adult congenital heart disease, biomarkers, survival, NT-proBNP, prognosis

## Abstract

**Background:**

The adult congenital heart disease (ACHD) population is growing and risk prediction is important to predict adverse outcome and consult patients during their lifecourse.

**Objectives:**

This study aims to describe the long-term prognostic value of blood biomarkers in ACHD.

**Methods:**

In this prospective observational cohort study, 602 patients with moderate or complex ACHD were included (median age 32.5 years [IQR: 24.7-41.2], 42% female, 90% New York Heart Association I). N-terminal pro-brain natriuretic peptide (NT-proBNP), high-sensitive-troponin T, growth differentiation factor 15, high-sensitive-C-reactive protein, suppression of tumorigenicity-2 and galectin-3, as well as full blood count, renal function, LDL, and HDL were measured. Cox models were applied to relate the selected biomarkers with the primary end point of all-cause mortality and secondary end point of mortality or heart failure. Standardized HRs adjusted for relevant prognostic factors, including age, sex, and complexity of diagnosis, were reported.

**Results:**

Abnormal biomarker levels were present in 424 (70.4%) patients. During a median follow-up of 10.1 years, 41 (6.8%) patients died and 81 (13.5%) developed heart failure. Associations were observed between the primary and secondary end point and red cell distribution width, NT-proBNP, and growth differentiation factor 15. In a multibiomarker model, only NT-proBNP remained associated with mortality (HR: 2.74; 95% CI: 2.01-3.74). NT-proBNP significantly improved the C-statistic of the clinical prediction model (0.85-0.92). Based on NT-proBNP alone, low-risk patients could be identified. Patients with NT-proBNP <76 ng/L showed a 10-year heart failure-free survival of 98.5%.

**Conclusions:**

Blood biomarkers have prognostic value in ACHD. NT-proBNP improves risk prediction and is able to identify low-risk patients. Its routine use should be implemented in ACHD.

Congenital heart disease is the most prevalent congenital defect with a birth prevalence of 9 per 1000 live births.^1^ Improvements in cardiac surgery and intensive care over the past decades drastically improved the prognosis of patients with congenital heart disease, with now more than 90% of patients surviving into adulthood.^2^^,^^3^ Consequently, the prevalence of adult congenital heart disease (ACHD) is increasing and this increase is expected to continue.^4^ Despite this prospect of increased survival, complications during adulthood pose a particular challenge. Throughout their life course, patients will be confronted with the sequelae of their disease. Residual cardiac lesions, arrhythmias, and heart failure require lifelong surveillance and often new interventions are needed. Risk stratification is crucial to identify patients at high risk of complications and, if possible, plan timely treatment (medication or (re)intervention).

Patients with ACHD show a high health care utilization that increases even further with age.^5^^,^^6^ This poses an important burden to primarily the patient, but also to our health care system. To keep health care manageable for the continuously growing population of ACHD patients, long-term risk prediction is of great importance. Short-term risk prediction is important for timing interventions and changing treatment strategies. But this may not be sufficient to guide long-term treatment strategies. In patients with a low long-term risk of adverse events, the follow-up intervals can be increased. Additionally, considering that the population of patients with ACHD is relatively young, long-term risk prediction makes their future prospects less unsure, especially for important decisions such as life planning and family planning.

Blood biomarkers play an important role in congestive heart failure in general cardiology.^7^ Various blood biomarkers may represent different pathophysiological mechanisms related to late complications in ACHD. N-terminal pro-brain natriuretic peptide (NT-proBNP), indicative of myocardial stress, has been a cornerstone in general cardiology for long. In ACHD too, NT-proBNP has shown to be an important predictor of adverse outcome.^8^, ^9^, ^10^ Increasing NT-proBNP levels during follow-up are associated with heart failure and mortality.^10^ In our research group, other prognostic biomarkers have been identified, including high-sensitive (hs) troponin T, growth differentiation factor 15 (GDF-15), galectin-3, hs-C-reactive protein (hs-CRP), red cell distribution width (RDW), and suppression of tumorigenicity-2 (ST-2).^8^^,^^11^, ^12^, ^13^, ^14^ Neurohormonal biomarkers such as norepinephrine, epinephrine, endothelin-1, renin, and aldosterone are associated with clinical variables of heart failure in the ACHD population.^15^ Cytokines representing the inflammatory pathway have shown to be elevated in patients with ACHD and show correlations with heart failure.^16^^,^^17^

The current study is based on our single-center cohort of adults with congenital heart disease. The name of our study is Biocon (BIOmarkers in CONgenital heart disease). Several manuscripts have been published based on this cohort.^8^, ^9^, ^10^, ^11^, ^12^, ^13^^,^^18^, ^19^, ^20^, ^21^ These and other studies investigating biomarkers in ACHD have a relatively short follow-up time. The evidence regarding the role of blood biomarkers on the long-term follow-up remains limited. The aim of this study was to investigate various blood biomarkers in the same cohort of patients with ACHD, but now with a prospective long-term follow-up of 10 years.

## Methods

### Study design and study population

In this prospective observational cohort study, all eligible adult patients with moderate or complex congenital heart disease were approached and included between April 2011 and April 2013 during routine outpatient visits until the required sample size was reached. Patients with mild ACHD, impaired renal function (creatinine >200 μmol/L), who were <18 years old or pregnant were excluded. At baseline, all patients underwent physical examination by a cardiologist, 12-lead electrocardiogram, echocardiography, and venous blood sampling. Included patients underwent per protocol follow-up for a minimum of one visit per year during the first 4 years after inclusion. After that, patients visited the outpatient clinic once a year or every 2 years as clinically indicated. Patients were treated according to the guidelines of the European Society of Cardiology and the discretion of the physician.^22^^,^^23^ The study was approved by the medical ethical committee (MEC-2010-165) and performed according to the principles outlined in the Declaration of Helsinki. All patients provided written informed consent.

### Blood biomarkers

Venous blood sampling was obtained after 30 minutes of rest. Samples were transferred to the local clinical chemistry laboratory. The following biomarkers were measured directly in fresh samples: hemoglobin, hematocrit, mean corpuscular volume, RDW, urea, creatinine, hs-CRP, total cholesterol, HDL-cholesterol, LDL-cholesterol, and NT-proBNP. Additional tubes were collected for further biomarker analysis (CTAD 4.5 ml, SST II 8.5 ml, and EDTA 6 ml). Within 1 hour after blood sampling, these tubes were centrifuged at 2000g for 10 minutes. After that, the blood samples were aliquoted and stored at −80 °C for further biomarker analysis according to a standardized protocol for biospecimen collection, preparation, and storage to guarantee quality and consistency for biomarker analysis. The following biomarkers were measured using stored blood samples: ST-2, hs-troponin T, galectin-3, and GDF-15. For further analysis, we used the following biomarkers: hematocrit, RDW, creatinine, total cholesterol, NT-proBNP, hs-troponin T, GDF-15, hs-CRP, ST-2, and galectin-3.

Normal values were determined based on cutoff values used in our clinical chemistry laboratory or based on previous research of our group including biomarker determination in healthy volunteers.^8^^,^^11^, ^12^, ^13^ A detailed overview of the used assays, limits of detection, and normal values are shown for all biomarkers in Supplemental Table 1.

### Study end points

All-cause mortality was the primary end point. The secondary end point was a composite end point including all-cause mortality and heart failure. Heart failure was defined as signs or symptoms of heart failure requiring hospitalization or the start or intensification of diuretics or heart failure-specific medication. Events were assessed, blinded to any biomarker levels, by two experienced researchers (P.H. and A.vd.B.). Survival status was checked using municipal records. Data on survival were complete for 100% of the patients. Patients who did not reach the end point were right-censored on April 1, 2022.

To evaluate the clinical evolution over 10 years of ACHD, we calculated, in accordance with previous publications of this cohort, the arrhythmia-free survival (arrhythmias defined as symptomatic and documented or requiring treatment) and event-free survival (consisting of a composite of: all-cause mortality, heart failure, arrhythmias, thromboembolic events, cardiac hospitalizations, and (re)interventions) in addition to the primary and secondary end points. If a patient experienced more than one event at the same time, both events were scored (ie heart failure and arrhythmia).

### Statistical analysis

Continuous variables are presented as mean ± standard deviation or median (interquartile range) depending on the distribution. Categorical variables are presented as cases (percentage). All biomarker levels were transformed to obtain a normal distribution and standardized.

The Kaplan-Meier method was used to obtain estimates of cumulative end point free survival. Cox proportional hazards (PH) regression models were used to evaluate the associations between the selected biomarkers and the study end points. We ran separate models for each biomarker, while adjusting for clinically important parameters.^24^ For the primary end point, we included age, sex, New York Heart Association (NYHA) class, and complexity of diagnosis as covariates. For the secondary end point, we included age, sex, NYHA class, venous oxygen saturation (<90%), cardiac medication use, complexity of diagnosis, and the presence of sinus rhythm taking into account 1 degree of freedom for a minimum of 10 events. To evaluate long-term value of the respective biomarkers, additional models were created based on patients that were free of the primary or secondary end point after 3 years of follow-up. HRs are reported per standard deviation increase as biomarker levels were standardized.

Furthermore, we applied a stepwise approach to construct multibiomarker models for the prediction of the primary and secondary end points. Nonlinearity was evaluated using natural cubic splines. The biomarkers that were significantly (*P* < 0.0051 according to the Sidak equation for multiple testing) associated with the study end points in multivariable Cox models entered a multibiomarker model in addition to the aforementioned clinical factors. Backward selection was then performed and the final model only included significant biomarkers. To evaluate the added predictive value of these biomarkers, the C-statistic of the model with biomarkers and the model with clinical factors only were calculated. Cox models were compared using the likelihood ratio test. The PH assumption of all Cox models was evaluated using Schoenfeld residuals and no violations were observed. Missing values were imputed for the Cox regression models using multiple imputation via chained equations (25 iterations, 20 imputed data sets generated). An overview of the variables used for imputation and the proportion of missingness is shown in Supplemental Table 2.

Statistical analysis was performed using SPSS (IBM Corp. Released 2017, IBM SPSS Statistics for Windows, Version 25.0. Armonk, NY: IBM Corp.) and R (R Core Team (2017). R: A language and environment for statistical computing. R Foundation for Statistical Computing, Vienna, Austria. URL https://www.R-project.org/) using the packages ‘survival,’ ‘ggplot2,’ ‘mice.’

## Results

### Patient population

A total of 602 patients with moderate or complex ACHD were included in this study. A flowchart of study inclusion was published previously.^8^ Baseline characteristics are presented in Table 1. The median age was 32.5 years (IQR: 24.7-41.2) and 57.8% were male. Surgical repair was performed in 90.9% of patients at a median age of 3.7 years (IQR: 0.8-11.9). An overview of the specific heart defects is detailed in Supplemental Table 3. The vast majority of patients were asymptomatic and in NYHA class I at inclusion. Sinus rhythm was present in 89.9% of patients. Of all patients, 71 (11.8%) used diuretics. The function of the systemic ventricle was normal in 59.5% of patients and mildly impaired in 35.7%. Median NT-proBNP levels were mildly elevated (128.5 ng/L). Profound differences were observed in biomarker levels between patients with moderate and complex heart disease. Higher levels of hemoglobin, hematocrit, RDW, urea, NT-proBNP, troponin T, GDF-15, ST-2, and galectin-3 were found in complex ACHD. Median NT-proBNP levels were 94.7 ng/L (IQR: 47.4-201.3) and 241.9 (IQR: 132.8-514.2) ng/L in, respectively, moderate and complex ACHD. Troponin T levels were also higher among patients with complex ACHD compared to moderate ACHD (4.0 [IQR: 2.1-6.7] ng/L vs 5.5 [IQR: 2.1-8.3] ng/L).

**Table 1:** Baseline Characteristics

	Complete Cases, n (%)	All Patients (N = 602)	Moderate ACHD (n = 417)	Complex ACHD (n = 185)	*P* Value
Clinical Parameters					
Age, years	602 (100)	32.5 [24.7-41.2]	33.0 [24.4-42.7]	31.9 [25.0-38.5]	0.179
Sex, male (%)	602 (100)	348 (57.8)	244 (58.5)	104 (56.2)	0.599
Initial repair (%)	602 (100)	547 (90.9)	377 (90.4)	170 (91.9)	0.213
Age at surgical repair, years	546 (90.7)	3.7 [0.8-11.9]	5.0 [0.8-15.5]	2.5 [0.7-6.5]	<0.001
Device any (%)	591 (98.2)	57 (9.6)	23 (5.5)	34 (18.4)	<0.001
BMI, kg/m^2^	599 (99.5)	24.2 [21.7-27.0]	24.5 [21.9-27.5]	23.5 [21.3-26.1]	0.026
Heart rate, bpm	594 (98.7)	72 [65-82]	72 [65-82]	72 [65-83]	0.923
Systolic blood pressure, mm Hg	591 (98.2)	125 [115-135]	126 [116-137]	122 [113-132]	0.005
O_2_ saturation <90% (%)	558 (92.7)	17 (3.0)	1 (0.3)	16 (9.4)	<0.001
NYHA class	602 (100)				<0.001
I (%)		541 (89.9)	400 (95.9)	141 (76.2)	
II (%)		56 (9.3)	15 (3.6)	41 (22.2)	
III (%)		5 (0.8)	2 (0.5)	3 (1.6)	
IV (%)		0 (−)	0 (−)	0 (−)	
Cardiac medication use, any	602 (100)	212 (35.2)	126 (30.2)	86 (46.5)	
ACE inhibitor (%)		89 (14.8)	50 (12.0)	39 (21.1)	0.004
Beta-blocker (%)		90 (15.0)	58 (13.9)	32 (17.3)	0.282
Calcium antagonist (%)		16 (2.7)	11 (2.7)	5 (2.7)	0.964
Diuretic (%)		71 (11.8)	30 (7.2)	41 (22.2)	<0.001
Hydrochlorothiazide (%)		13 (18.3)	13 (43.3)	0 (−)	
Loop diuretics (%)		58 (81.7)	17 (56.7)	41 (100)	
History of supraventricular arrhythmias, any	602 (100)	100 (16.6)	40 (9.6)	60 (32.4)	<0.001
Atrial fibrillation/flutter		66 (11.0)	30 (7.2)	36 (19.5)	
High-grade AV block		20 (3.3)	6 (1.4)	14 (7.6)	
Ectopic atrial rhythm		9 (1.5)	4 (1.0)	5 (2.7)	
Other		5 (0.8)	0 (−)	5 (2.7)	
History of heart failure	602 (100)	20 (3.3)	10 (2.4)	10 (5.4)	0.057
Electrocardiography					
Rhythm	602 (100)				<0.001
Sinus (%)		521 (86.5)	376 (90.2)	145 (78.4)	
Paced (%)		44 (7.3)	21 (5.0)	23 (12.4)	
Atrial fibrillation (%)		15 (2.5)	8 (1.9)	7 (3.8)	
Other (%)		22 (3.7)	12 (2.9)	10 (5.4)	
QRS duration, msec	558 (92.7)	112 [100-137]	110 [98-136]	116 [102-141]	0.050
Echocardiography					
Left atrial volume, mL/m^2^	432 (71.8)	20.5 [15.4-29.1]	40.5 [28.3-55.5]	35.1 [30.0-55.2]	0.594
LV end-diastolic volume, mL	410 (68.1)	115.8 [93.3-143.6]	112.9 [91.0-140.5]	148.7 [119.7-174.6]	<0.001
LV end-systolic volume, mL	409 (67.9)	49.2 [38.5-63.5]	47.8 [37.7-61.5]	70.9 [56.3-87.8]	<0.001
RV end-diastolic area, cm^2^	391 (65.0)	30.0 [23.6-38.2]	26.2 [21.9-33.1]	38.7 [33.2-47.4]	<0.001
RV end-systolic area, cm^2^	390 (64.8)	18.2 [12.6-25.6]	15.0 [11.7-20.0]	28.7 [22.7-33.8]	<0.001
E/A ratio	440 (73.1)	1.5 [1.2-2.0]	1.5 [1.2-2.0]	1.6 [1.3-2.1]	0.102
E’wave, m/s	395 (65.6)	8.0 [6.5-9.6]	8.0 [6.6-9.7]	7.3 [5.8-8.9]	0.031
E/E’ ratio	388 (64.5)	10.3 [8.1-14.1]	10.0 [8.1-13.8]	11.8 [8.5-15.2]	0.126
LV ejection fraction, %	409 (67.9)	56 [52-60]	57 [53-61]	51 [47-55]	0.008
RV fractional area change, %	390 (64.8)	38.3 ± 11.3	42.5 ± 9.1	28.5 ± 9.8	<0.001
Systemic ventricular function	602 (100)				<0.001
Normal (%)		302 (50.2)	278 (66.8)	24 (13.0)	
Mildly impaired (%)		212 (35.2)	122 (29.3)	90 (48.6)	
Moderately impaired (%)		69 (11.5)	10 (2.4)	59 (31.9)	
Severely impaired (%)		18 (3.1)	6 (1.4)	12 (6.5)	
Estimated RA pressure	423 (70.3)				0.019
5 mm Hg (%)		374 (88.4)	276 (91.1)	98 (81.7)	
10 mm Hg (%)		22 (5.2)	10 (3.3)	12 (10.0)	
15 mm Hg (%)		21 (5.0)	14 (4.6)	7 (5.8)	
20 mm Hg (%)		6 (1.4)	3 (1.0)	3 (2.5)	
Blood biomarkers					
Hemoglobin, mmol/L	592 (98.3)	9.3 [8.6-9.8]	9.2 [8.5-9.7]	9.4 [8.8-10.1]	<0.001
Hematocrit, L/L	592 (98.3)	0.44 [0.41-0.46]	0.43 [0.41-0.46]	0.44 [0.42-0.47]	<0.001
MCV, fL	592 (98.3)	88 [85-90]	88 [85-90]	89 [86-91]	0.019
RDW, %	592 (98.3)	13.0 [12.6-13.6]	12.9 [12.5-13.5]	13.3 [12.7-14.0]	<0.001
Urea, mmol/L	598 (99.3)	5.1 [4.3-6.1]	5.0 [4.3-6.0]	5.3 [4.3-6.2]	0.047
Creatinine, μmol/L	598 (99.3)	75 [67-84]	76 [67-84]	75 [67-83]	0.356
eGFR, ml/min/1.73 m^2^	598 (99.3)	90 [82-90]	90 [83-90]	90 [81-90]	0.718
Total cholesterol, mmol/L	523 (86.9)	4.7 [4.1-5.4]	4.8 [4.1-5.5]	4.5 [4.0-5.2]	0.036
LDL, mmol/L	523 (86.9)	2.85 [2.38-3.52]	2.9 [2.4-3.6]	2.8 [2.3-3.3]	0.155
HDL, mmol/L	523 (86.9)	1.35 [1.13-1.61]	1.4 [1.2-1.6]	1.3 [1.0-1.5]	<0.001
NT-proBNP, ng/L	595 (98.8)	128.5 [57.5-281.6]	94.7 [47.4-201.3]	241.8 [132.8-514.2]	<0.001
hs-Troponin T, ng/L	589 (97.8)	4.3 [1.5-7.2]	4.0 [2.1-6.7]	5.5 [2.1-8.3]	<0.001
GDF-15, ng/L	589 (97.8)	618 [487-866]	594 [476-785]	682 [507-1110]	<0.001
hs-CRP, mg/L	591 (98.2)	1.5 [0.6-3.5]	1.3 [0.6-3.5]	1.7 [0.7-3.5]	0.363
ST-2, ng/mL	600 (99.7)	24.1 [17.9-32.1]	23.0 [17.2-31.6]	28.9 [19.7-33.7]	0.008
Galectin-3, ng/mL	601 (99.8)	12.6 [10.8-14.6]	12.4 [10.7-14.3]	13.2 [11.2-15.5]	0.002

Values are presented as mean ± SD or median [IQR] for continuous variables or N (%) for categorical variables.

ACE = angiotensin converting enzyme; ACHD = adult congenital heart disease; AV-block = atrioventricular block; BMI = body mass index; eGFR = estimated glomerular filtration rate; HDL = high density lipoprotein; LDL = low density lipoprotein; LV = left ventricle; MCV = mean corpuscular volume; NYHA = New York Heart Association; RA = right atrium; RV = right ventricle; hs-CRP = high-sensitive C-reactive protein; GDF-15 = growth differentiation factor 15; NT-proBNP = N-terminal pro-brain natriuretic peptide; RDW = red cell distribution width; ST-2 = suppression of tumorigenicity-2.

### Survival

Survival status was available in 100% and follow-up with regard to the other end points was complete in 579 (96.2%) patients. During a median follow-up of 10.1 years (IQR: 9.7-10.5), 41 (6.8%) patients died, 81 (13.5%) developed heart failure, 215 (35.9%) were hospitalized for cardiac reasons, 181 (30.1%) had an arrhythmia, 40 (6.7%) had a thromboembolic event, and 190 (31.7%) underwent a (re)intervention. A detailed overview of all events is shown in Supplemental Table 4. The secondary end point of death or heart failure was reached in 99 (16.4%) patients. In our cohort, we observed a 10-year cumulative survival of 93.3% (95% CI: 91.3-95.4%), heart failure-free survival of 83.7% (95% CI: 80.8-86.8%), arrhythmia-free survival of 66.8% (95% CI: 63.1-70.8%), and cardiovascular event-free survival of 49.1% (95% CI: 45.2-53.4%) (Supplemental Figure 1).

### Blood biomarkers

**Figure 1:**
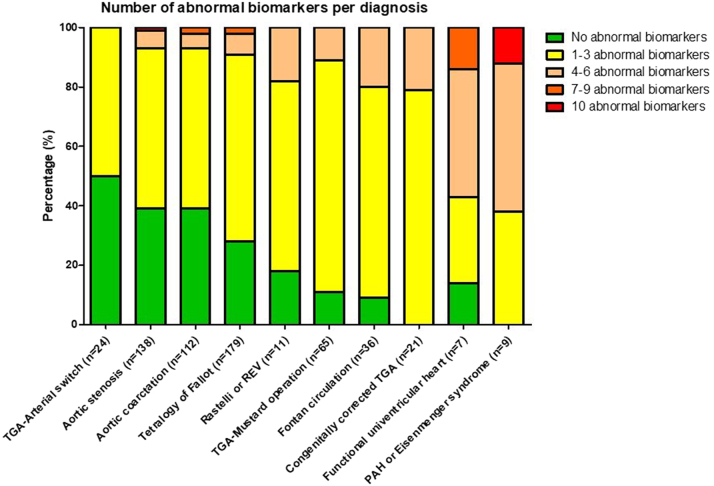
Number of Abnormal Biomarkers per Diagnosis Included Biomarkers: Hematocrit, RDW, Creatinine, Total Cholesterol, NT-proBNP, hsTnT, GDF-15, hs-CRP, ST-2, and Galectin-3. Cutoff Values as Defined in Supplemental Table 1. hs-CRP = high-sensitive C-reactive protein; GDF-15 = growth differentiation factor 15; NT-proBNP = N-terminal pro-brain natriuretic peptide; PAH = pulmonary arterial hypertension; RDW = red cell distribution width; REV = réparation à l’etage ventriculaire; ST-2 = suppression of tumorigenicity-2; TGA = transposition of the great arteries.

One or more abnormal biomarker levels were present in 424 (70.4%) patients at baseline. Figure 1 illustrates the number of abnormal biomarkers per diagnosis. Patients after arterial switch repair of transposition of the great arteries, aortic stenosis, and aortic coarctation presented with the least abnormal biomarker levels. The highest number of abnormal biomarkers was found in patients with a congenitally corrected transposition of the great arteries, functionally univentricular heart and pulmonary arterial hypertension or Eisenmenger syndrome. Survival and heart failure-free survival were worse with increasing number of abnormal biomarkers (Supplemental Figure 2). Correlations between blood biomarkers were shown in Supplemental Figure 3.

Multivariable Cox PH regression showed significant associations between the primary end point and higher levels of RDW (HR: 1.60; 95% CI: 1.26-2.05), NT-proBNP (HR: 3.74; 95% CI: 2.46-5.64), hs-troponin T (HR: 2.12; 95% CI: 1.58-2.83), GDF-15 (HR: 3.89; 95% CI: 2.20-6.89), and ST-2 (HR: 1.77; 95% CI: 1.23-2.53). Crude estimates are shown in Supplemental Table 5. Hematocrit, creatinine, total cholesterol, hs-CRP, and galectin-3 did not show any significant associations. Hematocrit and creatinine showed a nonlinear association with the primary and the secondary end point. Including the nonlinear effects in the regression models did not alter the significance of our findings (Supplemental Tables 6 and 7). The secondary end point of death or heart failure was associated with RDW (HR: 1.40; 95% CI: 1.19-1.67), NT-proBNP (HR: 2.97; 95% CI: 2.16-4.06), hs-troponinT (HR: 1.35; 95% CI: 1.09-1.67), and GDF-15 (HR: 1.80; 95% CI: 1.28-2.56). Supplemental Table 8 shows a subgroup analysis per diagnostic group. When only patients who did not reach the end point within the first 3 years were included, RDW and ST-2 were not significantly associated with the primary end point anymore (Supplemental Table 9). For the secondary end point, no significant associations were found between RDW and troponin T (Supplemental Table 9).

### Multibiomarker model

In the multibiomarker model for the primary end point of all-cause mortality, NT-proBNP was the only blood biomarker that remained significantly associated with a HR of 3.74 (95% CI: 2.46-5.64) per standard deviation increase (Table 2). In the multibiomarker model for the secondary end point, NT-proBNP and RDW remained significant predictors with HRs of, respectively, 2.74 (95% CI: 2.01-3.74) and 1.29 (95% CI: 1.08-1.55).

**Table 2:** Standardized Hazard Ratios for Blood Biomarkers and Primary and Secondary End Point

Biomarker	Death	*P* Value^a^	Death or Heart Failure	*P* Value^a^
	HR (95% CI)		HR (95% CI)	
Hematocrit^b^	0.93 (0.67-1.30)	0.659	0.74 (0.59-0.93)	0.018
RDW	1.60 (1.26-2.05)	<0.001	1.40 (1.19-1.67)	<0.001
Creatinine^b^	1.35 (1.03-1.79)	0.033	0.91 (0.74-1.12)	0.385
Total cholesterol	0.70 (0.49-1.00)	0.050	0.88 (0.71-1.09)	0.253
NT-proBNP	3.74 (2.46-5.64)	<0.001	2.97 (2.16-4.06)	<0.001
Troponin T	2.12 (1.58-2.83)	<0.001	1.35 (1.09-1.67)	0.006
GDF-15	3.89 (2.20-6.89)	<0.001	1.80 (1.28-2.56)	0.001
CRP	1.63 (1.16-2.29)	0.006	1.32 (1.07-1.65)	0.011
ST-2	1.77 (1.23-2.53)	0.002	1.25 (0.98-1.58)	0.077
Galectine-3	1.27 (0.91-1.77)	0.111	1.00 (0.81-1.23)	0.990

Each Model Is Adjusted for Age (Years), Sex, Diagnosis (Moderate ACHD (0) Vs Complex ACHD (1)), Saturation, Use of Cardiac Medication (Yes/No), Sinus Rhythm (Yes/No), and Systemic Ventricular Function (0-3).

Abbreviations as in Table 1.

a *P* values <0.0051 are considered statistically significant according to Sidak’s equation for multiple testing.

b Biomarkers had a nonlinear association with the end points. Including the nonlinear term in the regression model did not alter the significance of the relation between the biomarker and the primary and secondary end point.

Table 3 shows the C-statistics for the clinical model and the models including NT-proBNP and/or RDW. The isolated clinical model already showed a very good discriminability for both the end points of death and death or heart failure. Addition of NT-proBNP to the model significantly increased the C-statistic for survival (0.85-0.92) and heart failure-free survival (0.85-0.88). The addition of RDW to the model for death or heart failure including NT-proBNP showed a limited improvement, albeit significant, for the prediction of the secondary end point (0.88-0.89). The C-statistic for the primary end point including both NT-proBNP and RDW was not calculated since RDW was not a significant predictor for the primary end point in the multibiomarker model.

**Table 3:** C-Statistic for the Clinical Model (Consisting of: Age, Sex, Complexity of Diagnosis, Saturation, Use of Cardiac Medication, Sinus Rhythm and Systemic Ventricular Function) and the Addition of NT-proBNP and Red Cell Distribution Width

Model	Death	Death or Heart Failure
	C-Statistic	C-Statistic
Clinical model	0.85	0.85
Clinical model with addition of NT-proBNP	0.92	0.88
Clinical model with addition of NT-proBNP and RDW	-	0.89

Abbreviations as in Table 1.

**Figure 2:**
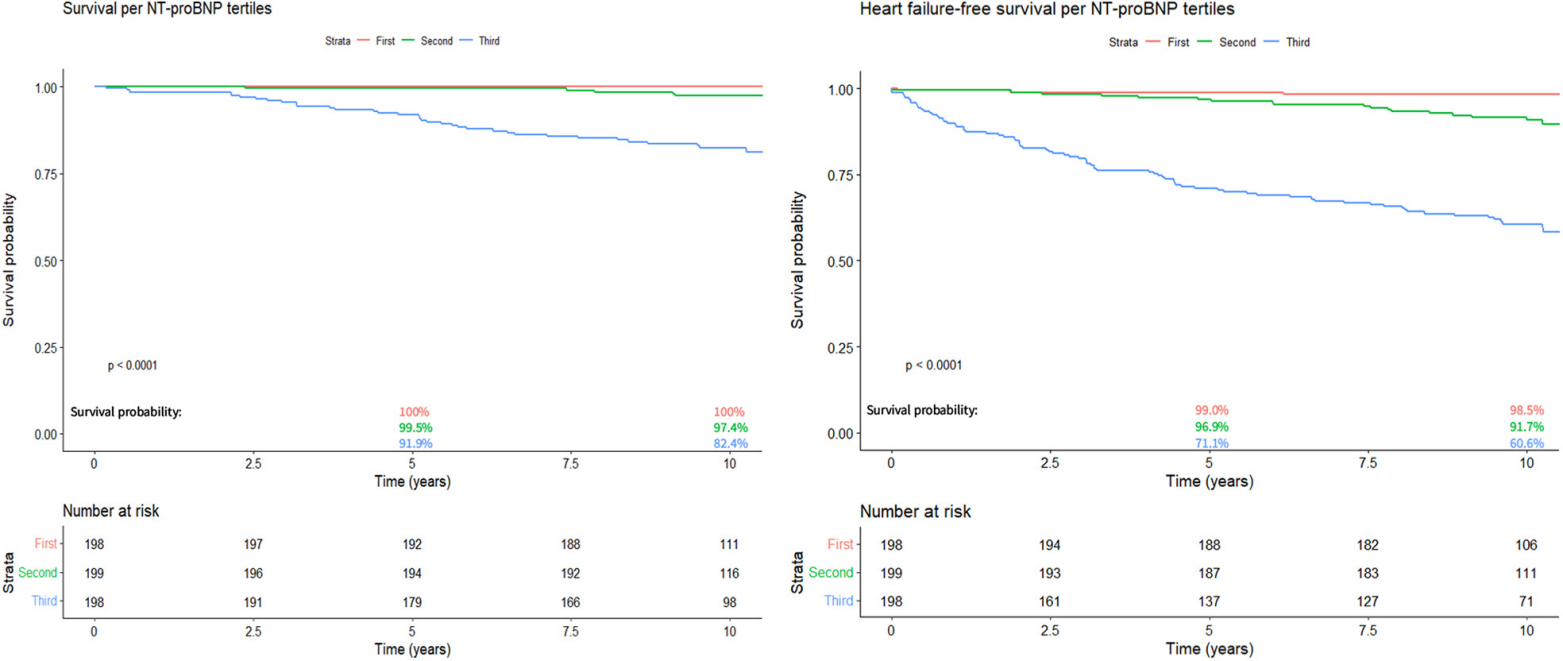
Survival and Heart Failure-Free Survival According to NT-proBNP Tertile First Tertile: 3.4 to 76.1 ng/L, Second Tertile: 80.0 to 208.9 ng/L, Third Tertile: 213.1 to 6993.9 ng/L. Abbreviation as in Figure 1.

Based on the prominent role of NT-proBNP, Figure 2 shows that in patients within the first and second tertile, survival and heart failure-free survival are near excellent. At 10 years, survival was 100% for patients within the first tertile and 97.4% within the second tertile of NT-proBNP. In the third tertile, survival was 82.4%. Heart failure-free survival at 10 years was also excellent in the first tertile (98.5%), slightly lower in patients in the second tertile (91.7%) and distinctively lower in the last tertile (60.6%). Supplemental Tables 10 and 11 show baseline characteristics and congenital diagnosis per tertile.

## Discussion

In this prospective observational cohort study with 10 years of follow-up, we investigated the long-term prognostic value of various blood biomarkers. In our relatively young population of patients with ACHD, the majority had at least one abnormal blood biomarker. In more complex ACHD, even a bigger proportion had at least abnormal blood biomarkers and the number of abnormal biomarkers was higher too. In multivariable analysis RDW, NT-proBNP, troponin T, GDF-15, and ST-2 were associated with death or heart failure. In a multibiomarker model, NT-proBNP remained the most prominent predictive blood biomarker. Patients with a low NT-proBNP had an excellent 10-year (heart failure-free) survival (Central Illustration).

**Central Illustration:**
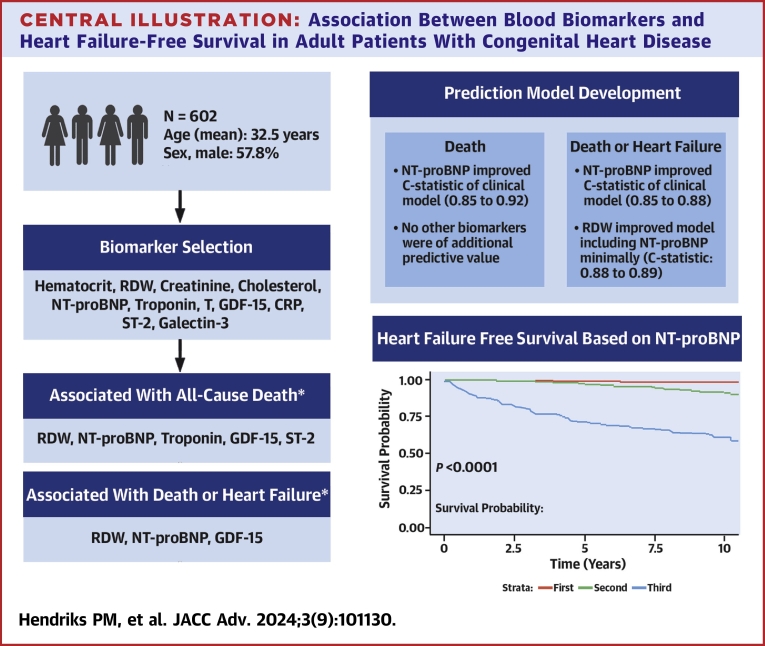
Association Between Blood Biomarkers and Heart Failure-Free Survival in Adult Patients With Congenital Heart Disease Multibiomarker Model, Including All Biomarkers. Clinical Model: Age, Sex, Complexity of Diagnosis, Saturation, Cardiac Medication, Presence of Sinus Rhythm, Systemic Ventricular Function. ∗Adjusted for: Age, Sex, Complexity of Diagnosis, Saturation, Cardiac Medication Presence of Sinus Rhythm, Systemic Ventricular Function. Abbreviations as in Figure 1.

NT-proBNP is a cornerstone of contemporary cardiology and is well established as a biomarker, especially in heart failure. It is a preprohormone of BNP and is released as a response to increased ventricular wall stress. In ACHD, elevated BNP levels have been observed as well, even in patients that are considered clinically stable.^8^^,^^25^ It can be hypothesized that even in patients that are considered clinically stable, ongoing increased myocyte stress results in elevation of NT-proBNP levels. Previous studies established the use of NT-proBNP as an important predictor for adverse outcome and mortality in ACHD in short-term follow-up.^8^^,^^26^ NT-proBNP also shows a rising trend shortly before the development of heart failure or mortality.^10^ This study demonstrated that NT-proBNP does not only provide prognostic information on the short term or just before the occurrence of an adverse event, it is also a strong predictor of long-term clinical outcome. Even in a clinically stable state of disease, NT-proBNP provides information that is relevant in the long-term risk stratification of patients with ACHD. We performed a subgroup analysis and this showed that NT-proBNP showed significant associations with all diagnostic groups and therefore is able to provide prognostic information in all congenital heart defects. Other biomarkers showed varying associations between different diagnostic groups (ie hsTnT, GDF-15, and RDW).

### Multibiomarker approach

The multibiomarker models showed that, complementary to clinical data and NT-proBNP, RDW too was significantly associated with heart failure-free survival. RDW is a hematological marker of anisocytosis. It has been associated with morbidity and mortality in a vast array of pathology including cardiovascular disease and heart failure.^27^, ^28^, ^29^ In two large contemporary cohorts of symptomatic heart failure patients, the CHARM program and Duke cohort, RDW was one of the most powerful predictors of morbidity and mortality when compared to several clinical risk predictors.^30^ In a previous study of our ACHD cohort, RDW showed a modest increase in predictive value additional to a model including NT-proBNP.^12^ Despite most of the patients being in the normal range of RDW reference values, it was observed that patients with an event had higher RDW levels. Longitudinal analysis of repeated RDW measurements did not demonstrate a distinct change in RDW prior to an event.^12^ It remains unclear whether RDW plays a pathophysiological role or is an epiphenomenon on the prognostic pathway. RDW showed a weak correlation with NT-proBNP, but no significant correlations with other hematologic parameters. RDW could be an interesting blood biomarker especially as it is widely available and inexpensive. However, there are some caveats that should be taken into account with regard to RDW, especially the specificity of RDW is expected to be modest as its associations are not limited to cardiovascular disease which impedes clinical decision-making based on this marker. In this long-term follow-up study, RDW too was significantly associated with heart failure-free survival in addition to NT-proBNP. However, the additional value of RDW to a prediction model already including NT-proBNP was limited.

Several biomarkers such as hs-troponin T, CRP, GDF-15, and ST-2 showed a significant association with the end points. This could indicate that complex and possibly less well understood systemic mechanisms play a major role in the prognosis of ACHD. We also observed worse survival with increasing numbers of abnormal biomarkers. However, when adjusted for NT-proBNP, these blood biomarkers provided little additional predictive information.

### Strengths and limitations

There are several strengths and limitations to this study. This study includes a broad range of blood biomarkers but could not include all potentially relevant blood biomarkers. The selection of biomarkers was based on clinically available blood biomarkers as well as novel blood biomarkers. Some blood biomarkers were measured directly in the clinical chemistry laboratory. Treating physicians had access to these results and this could have influenced treatment and follow-up strategies. However, the biomarkers for which this applied, are part of routine clinical care and it would therefore not be ethical to blind physicians to these results. Furthermore, only moderate or complex ACHD was included in this cohort limiting the external validity to simple ACHD. The primary and secondary end point of the current study differed from the end points used in previous publications about this cohort (event-free survival including death, heart failure, cardiac hospitalization, (re)intervention, and thromboembolic events), therefore the associations with the end point cannot directly be compared to previous publications. However, including all these different end points in a composite end point might dilute or distort the underlying effects and effect sizes of different biomarkers. Therefore, we chose to use the end points death and the composite of death or heart failure. Despite these limitations, there are important strengths to this study. To our knowledge, this is the largest study investigating a broad range of both established and novel blood biomarkers in patients with moderate or complex ACHD. Furthermore, this study provided a median prospective follow-up of more than 10 years with minimal loss to follow-up. This is to our knowledge, the first study investigating the negative predictive value of NT-proBNP and its clinical consequences in ACHD enabling biomarker-based clinical decision-making.

## Clinical perspectives

The growing population of ACHD patients and especially those with complex defects will make future management challenging. Health care utilization and hospitalization are high and continue to increase with age.^5^^,^^6^ Reliable and reproducible risk stratification is of great importance in order to identify high-risk patients, so timely intervention can be offered. On the other hand, identification of low-risk patients can allow for larger follow-up intervals and fewer investigations, reducing both the burden for the patient and for health care systems.

This study demonstrated that a single NT-proBNP measurement could identify patients at very low risk for mortality or heart failure. Therefore, NT-proBNP is a valuable biomarker to not just identify patients with high risk, but also those at very low risk. This could aid both doctor and patient in the clinical follow-up. However, it should be taken into account that patients may face multiple other problems besides death or heart failure that were not taken into account in our analysis. Furthermore, in the ACHD population in particular it is of crucial importance to counsel patients regarding, ie, pregnancy, contraconception, exercise, comorbidities, and monitor neurocognitive and psychological health. NT-proBNP provides a good estimation of the cardiac risk and therefore, the use of NT-proBNP should be implemented as standard in clinical ACHD care. The added value of other blood biomarkers seems to be limited based on our results, although RDW does deserve some more study given the fact that it is widely available and appears to be carrying prognostic value, despite that this is not additionally to NT-proBNP and many clinical and imaging parameters in our study.

## Conclusions

During a 10-year prospective follow-up period, we observed a cumulative survival of 93%, heart failure-free survival of 83%, arrhythmia-free survival of 67%, and cardiovascular event-free survival of 49% in patients with moderate or complex ACHD. Blood biomarkers were able to predict 10-year clinical outcomes in these patients. NT-proBNP was the most prominent blood biomarker and showed a strong association with both all-cause mortality and heart failure-free survival. Based on the sole use of NT-proBNP, low-risk patients could be identified. The use of NT-proBNP should be implemented in clinical care.

Perspectives **COMPETENCY IN MEDICAL KNOWLEDGE:** Blood biomarkers provide prognostic information additional to widely used clinical and echocardiographic parameters. RDW, NT-proBNP, and GDF-15 are associated with all-cause mortality and heart failure. NT-proBNP is the most important biomarker in improving risk prediction in a multibiomarker model in addition to clinical prognostic factors. Patients in the lowest NT-proBNP tertile have excellent survival and can be reassured.

**COMPETENCY IN PATIENT CARE:** The single use of NT-proBNP enables the identification of patients at very low risk for heart failure and mortality with a 10-year risk of 1.5%. Based on NT-proBNP measurements alone, follow-up intervals could be increased.

**TRANSLATIONAL OUTLOOK:** Although NT-proBNP provided important clinical information in long-term risk prediction, this does not imply that other blood biomarkers do not provide clinical important information. The use of these blood biomarkers, especially RDW, warrants further research.

## Funding support and author disclosures

This study was supported by a grant from the Dutch Heart Foundation, Den Haag, the Netherlands (grant number 2015T029). The authors have reported that they have no relationships relevant to the contents of this paper to disclose.
