# Supplementary material for: Blood Biomarkers Predict 10-Year Clinical Outcomes in Adult Patients With Congenital Heart Disease
Source: JACC Adv. 2024 Jul 27;3(9):101130. doi: 10.1016/j.jacadv.2024.101130 (PMC11327932; doi:10.1016/j.jacadv.2024.101130)
Supplement: Supplementary data [file mmc1.pdf]

Supplemental material

Supplemental table 1: Overview of used assay, lower limits of detection and normal values per analyzed biomarker.

Biomarker	Assay	Lower Limit of detection	Normal values
Hemoglobin	Sysmex XN-1000^{TM} Hematology Analyzer (Sysmex Europe GmbH, Norderstedt, Germany)	-	>8.6 mmol/L men<br>>7.5 mmol/L women
Hematocrit	Sysmex XN-1000^{TM} Hematology Analyzer (Sysmex Europe GmbH, Norderstedt, Germany)	-	0.4-0.5 L/L
MCV	Sysmex XN-1000^{TM} Hematology Analyzer (Sysmex Europe GmbH, Norderstedt, Germany)	-	80-100 fL
RDW	Sysmex XN-1000^{TM} Hematology Analyzer (Sysmex Europe GmbH, Norderstedt, Germany)	-	12-16 %
Urea	Commercial Colorimetric quantitative assay (Roche Diagnostics, Rotkreuz, Switzerland)	-	2.5-7.5 mmol/L
Creatinine	Commercial Colorimetric quantitative assay (Roche Diagnostics, Rotkreuz, Switzerland)	-	(65-)115 µmol/L
eGFR	-	-	>60 ml/min/1.72m^2
Total cholesterol	Commercial Colorimetric quantitative assay (Roche Diagnostics, Rotkreuz, Switzerland)	-	(2.9-)6.5 mmol/L
LDL	Commercial Colorimetric quantitative assay (Roche Diagnostics, Rotkreuz, Switzerland)	-	2.59-4.5 mmol/L
HDL	Commercial Colorimetric quantitative assay (Roche Diagnostics, Rotkreuz, Switzerland)	-	>1 mmol/L
NT-proBNP	Commercial Electrochemiluminescence immunoassay (Roche Diagnostics, Rotkreuz, Switzerland)	-	<15 ng/L
hsTnT	Commercial Electrochemiluminescence immunoassay (Roche Diagnostics, Rotkreuz, Switzerland)	5 ng/L	<14 ng/L
GDF-15	Precommercial Electrochemiluminescence immunoassay (Roche Diagnostics, Rotkreuz, Switzerland)	400 ng/L	<1109 ng/L
hsCRP	Commercial Immunoturbidimetric assay (Roche Diagnostics, Basel, Switzerland)	0.30 mg/L	<10 mg/L
ST-2	Presage ST2 assay (Critical Diagnostics, San Diego, California, USA)	2.4 ng/mL	<53 ng/mL
Galectin-3	ARCHITECT ci8200 analyser - galectin-3 assay (Abbott Diagnostics, Hoofddorp, The Netherlands)	4.0 ng/mL	<10.9 ng/mL

Supplemental table 2: variables used for imputation and proportion of missingness.

Covariate	Missing values<br>N (%)
**Age**	**0 (-)**
**Sex**	0 (-)
**Diagnosis**	0 (-)
**NYHA-class**	0 (-)
**Cardiac medication use**	0 (-)
**Heart rhythm**	0 (-)
**Systemic ventricular function**	0 (-)
**Occurrence of death**	0 (-)
**Occurrence of heart failure**	0 (-)
**Occurrence of arrhythmias**	0 (-)
**Galectin-3**	1 (0.17)
**ST-2**	2 (0.33)
**Creatinine**	4 (0.66)
**Urea**	4 (0.66)
**NT-proBNP**	7 (1.16)
**Hemoglobin**	10 (1.66)
**Hematocrit**	10 (1.66)
**MCV**	10 (1.66)
**RDW**	10 (1.66)
**hsCRP**	11 (1.83)
**GDF-15**	12 (1.99)
**hsTnT**	13 (2.16)
**Saturation**	44 (7.31)
**Total cholesterol**	79 (13.12)
**LDL cholesterol**	79 (13.12)
**HDL cholesterol**	79 (13.12)

Supplemental table 3: Diagnosis of patients at ACHD outpatient clinic
Abbreviations: ASD: atrial septal defect; REV: réparation à l'etage ventriculaire; TGA: transposition of the great arteries

**Diagnosis**	**Frequency N, (%)**
Tetralogy of Fallot	179 (29.7)
Aortic stenosis	138 (22.9)
Aortic coarctation	112 (18.6)
TGA-Mustard correction	65 (10.8)
Fontan circulation	36 (6.0)
TGA-Arterial switch	24 (4.0)
Congenitally corrected TGA	21 (3.5)
Rastelli/REV	11 (1.8)
Pulmonary arterial hypertension / Eisenmenger syndrome with underlying ASD	9 (1.5)
Functional univentricular heart	7 (1.2)

Supplemental table 4: Specification of events

Endpoints	Frequency N, (%)
All-cause mortality	41 (6.8)
End-stage heart failure	14 (34.2)
Cardiac arrest	6 (14.6)
Sudden, presumed cardiac	5 (12.2)
Other	16 (39.0)
Neoplastic	4 (25.0)
Infectious	2 (12.5)
Postoperative complication	2 (12.5)
Ischemic cerebrovascular accident	1 (6.3)
Multiple organ failure	1 (6.3)
Unknown	6 (37.5)
Heart failure	81 (13.5)
Cardiac hospitalization	214 (35.5)
Percutaneous intervention	68 (31.8)
Surgical intervention	59 (27.6)
Heart failure	27 (12.6)
Arrhythmia	17 (7.9)
Thromboembolic event	15 (7.0)
Other	28 (13.1)
Cardiac infections	13 (44.6)
Titration of medication	3 (10.7)
Syncope with no observed arrhythmia	2 (7.1)
Initiation of peritoneal dialysis (severe cardiorenal syndrome)	1 (3.6)
MINOCA	1 (3.6)
Inpatient screening for heart transplant	1 (3.6)
Digoxin intoxication	1 (3.6)
Suspect valvular thrombus	1 (3.6)
Multiple organ failure	1 (3.6)
Coronary angiography with 3-vessel disease	1 (3.6)
Inappropriate ICD-shock in AF with fast ventricular response	1 (3.6)
Atypical chest pain	1 (3.6)
Pacemaker replacement	1 (3.6)
Arrhythmia	181 (30.1)
Supraventricular arrhythmia, unspecified	41 (22.7)
Non-sustained ventricular tachycardia	38 (21.0)
Atrial fibrillation	38 (21.0)
Atrial flutter	37 (20.3)
Atrioventricular block	8 (4.4)
Sustained ventricular tachycardia	8 (4.4)
AV(N)RT	3 (1.7)
Ventricular extrasystoles (>10%)	3 (1.7)
Tachyarrhythmia, unspecified	3 (1.7)
Sinus arrest	2 (1.1)
Thromboembolic event	40 (6.7)
Ischemic cerebrovascular event	25 (62.5)
Pulmonary embolism	5 (12.5)
Myocardial infarction	5 (12.5)
Other	5 (12.5)
Superficial venous thrombosis	1 (20.0)
Retinal vein occlusion	1 (20.0)
Femoral artery thrombosis	1 (20.0)
Mesenteric artery thrombosis	1 (20.0)

Renal infarction	1 (20.0)
Reintervention	190 (31.7)
Surgical reintervention	83 (13.9)
Valve replacement	68 (82.0)
Valve repair	4 (4.8)
Aortic surgery	4 (4.8)
Other	7 (8.4)
Baffle stenosis repair	1 (14.3)
Defect closure	1 (14.3)
Tricuspid valve and baffle replacement	1 (14.3)
Bentall reoperation	1 (14.3)
Lateral tunnel placement Fontan	1 (14.3)
Bentall + ascending aorta hemiarch replacement	1 (14.3)
Replacement infected pacemaker	1 (14.3)
Percutaneous intervention	118 (19.7)
Ablation	39 (33.1)
Pacemaker or ICD implantation	32 (27.1)
Valve replacement	23 (19.5)
Coronary intervention	8 (6.8)
Valvular balloon dilatation	6 (5.1)
Defect closure	5 (4.2)
Aortic stenting	3 (2.5)
Other	2 (1.7)
Closure tunnel leaks	1 (50.0)
Pulmonary artery stenting	1 (50.0)

Supplemental table 5: Crude hazard ratios for primary and secondary endpoint per standard deviation increase

	**Death**		**Death or heart failure**	
	**Crude HR (95%-CI)**	**p-value**	**Crude HR (95%-CI)**	**p-value**
**Hematocrit**	1.13 (0.82-1.56)	0.443	1.00 (0.80-1.23)	0.987
**RDW**	1.82 (1.50-2.21)	<0.001	1.80 (1.57-2.05)	<0.001
**Creatinine**	1.64 (1.31-2.16)	<0.001	1.15 (0.94-1.41)	0.163
**Total Cholesterol**	0.91 (0.65-1.29)	0.604	1.07 (0.86-1.34)	0.541
**NT-proBNP**	4.97 (3.53-7.03)	<0.001	4.31 (3.46-5.37)	<0.001
**hs-Troponin T**	2.61 (2.11-3.22)	<0.001	1.99 (1.72-2.29)	<0.001
**GDF-15**	5.05 (3.13-8.08)	<0.001	3.57 (2.63-4.76)	<0.001
**Hs-CRP**	1.61 (1.18-2.20)	0.004	1.55 (1.27-1.90)	<0.001
**ST2**	1.66 (1.22-2.26)	0.002	1.23 (1.01-1.52)	0.038
**Galectine-3**	1.96 (1.50-2.57)	<0.001	1.80 (1.51-2.01)	0.004

Supplemental table 6: results of analysis of non-linear terms. P-values indicate the result of a likelihood ratio test comparing the model including the model including the non-linear effect to the nested model excluding this term. Non-significant p-values (>0.05) indicate that there is no improvement of the model including the respective non-linear or interaction term.

	**Death**	**Death or heart failure**
**Biomarker**	**p-value**	**P-value**
**Hematocrit**	0.004	0.082
**RDW**	0.919	0.294
**Creatinine**	0.011	0.008
**Total cholesterol**	0.214	0.037
**NT-proBNP**	0.120	0.202
**Troponin T**	0.788	0.564
**GDF-15**	0.072	0.856
**hsCRP**	0.715	0.993
**ST-2**	0.075	0.315
**Galectin-3**	0.382	0.373

Supplemental table 7: results of the cox-PH model including non-linear effects of hematocrit and creatinine with p-values per natural spline of the biomarker level. P-values <0.0051 are considered statistically significant according to Sidak's equation for multiple testing

	**Death p-value**	**Death or heart failure p-value**
**ns(hematocrit,1)**	0.021	0.020
**ns(hematocrit,2)**	0.070	0.279
**ns(hematocrit,3)**	0.070	0.290
**ns(creatinine,1)**	0.941	0.027
**ns(creatinine,2)**	0.712	0.053
**ns(creatinine,3)**	0.054	0.735

Supplemental table 8: subgroup analysis of combined diagnostic groups: systemic LV (aortic stenosis, aortic coarctation and TGA arterial switch), systemic right ventricle (ccTGA and TGA-Mustard correction), univentriculair heart and Fontan circulation, Tetralogy of Fallot/Rastelli/REV.

	**Systemic LV (n=274)**	**Systemic RV (n=86)**	**ToF/REV/Rastelli (n=190)**	**Univentricular and Fontan (n=43)**
**Biomarker**	**HR (95%-CI)**	**HR (95%-CI)**	**HR (95%-CI)**	**HR (95%-CI)**
Hematocrit	0.64 (0.31-1.31)	0.46 (0.29-0.71)	1.01 (0.57-1.78)	1.39 (0.80-2.41)
RDW	1.25 (0.77-2.04)	1.85 (1.35-2.55)	1.67 (1.20-2.33)	2.22 (1.27-3.88)
Creatinine	0.88 (0.56-1.40)	1.03 (0.57-1.85)	0.84 (0.53-1.35)	1.10 (0.53-2.28)
Total cholesterol	0.73 (0.44-1.20)	1.00 (0.59-1.68)	1.07 (0.66-1.72)	1.12 (0.56-2.22)
NT-proBNP	5.18 (2.68-10.03)	3.97 (2.08-7.58)	2.83 (1.74-4.59)	2.63 (1.12-6.20)
Troponin T	1.24 (0.79-1.94)	1.79 (1.18-2.72)	1.47 (1.03-2.10)	3.77 (1.44-9.85)
GDF-15	2.05 (0.96-4.38)	2.70 (1.35-5.37)	2.99 (1.52-5.85)	1.52 (0.60-3.86)
CRP	1.80 (1.07-3.05)	1.64 (1.03-2.63)	1.99 (1.29-3.06)	1.16 (0.58-2.32)
ST-2	0.65 (0.37-1.14)	1.04 (0.66-1.64)	1.71 (1.12-2.59)	2.77 (1.32-5.83)
Galectine-3	0.84 (0.55-1.28)	1.27 (0.80-2.01)	1.54 (1.01-2.36)	1.88 (0.99-3.56)

Supplemental table 9: Standardized hazard ratios for blood biomarkers and primary and secondary endpoint for patients who were free of the respective endpoint 3 years after inclusion. Each model is adjusted for age (years), sex, diagnosis (moderate ACHD (0) vs complex ACHD (1)), saturation, use of cardiac medication (yes/no), sinus rhythm (yes/no) and systemic ventricular function (0-3).

[a] P-values <0.0051 are considered statistically significant according to Sidak's equation for multiple testing.

	**Death**		**Death or heart failure**	
**Biomarker**	**HR (95%-CI)**	**p-value[a]**	**HR (95%-CI)**	**p-value[a]**
Hematocrit	0.93 (0.63-1.39)	0.724	1.11 (0.77-1.58)	0.571
RDW	1.50 (1.06-2.12)	0.025	1.26 (0.93-1.69)	0.123
Creatinine	1.16 (0.78-1.73)	0.443	1.08 (0.82-1.43)	0.558
Total cholesterol	0.62 (0.40-0.95)	0.030	0.77 (0.55-1.08)	0.133
NT-proBNP	4.47 (2.35-8.47)	<0.001	3.66 (2.36-5.70)	<0.001
Troponin T	1.85 (1.23-2.80)	0.006	1.27 (0.92-1.74)	0.143
GDF-15	3.68 (1.84-7.37)	<0.001	2.43 (3.91-1.51)	<0.001
CRP	1.48 (0.98-2.25)	0.060	1.17 (0.96-1.80)	0.092
ST-2	1.56 (1.01-2.38)	0.043	1.41 (1.02-1.95)	0.040
Galectine-3	1.15 (0.79-1.68)	0.451	0.98 (0.71-1.36)	0.913

Supplemental table 10: Baseline characteristics according to NT-proBNP tertile

	**NT-proBNP tertiles (n=595)**		
	**Tertile 1 (3.4-76.1 ngl/L, n=198)**	**Tertile 2 (80.0-208.9 ng/L, n=199)**	**Tertile 3 (213.1-6993.9 ng/L, n=198)**
**Clinical parameters**			
Age, years	27.8 [21.6-35.9]	30.7 [24.1-39.1]	39.2 [31.3-47.1]
Sex, male (%)	154 (77.8)	110 (55.3)	82 (41.4)
Initial repair (%)	170 (85.9)	183 (92.0)	187 (94.4)
Age at surgical repair, years	3.0 [0.6-13.2]	2.7 [0.6-9.5]	5.6 [1.8-15.3]
Device any (%)	7 (3.5)	53 (26.6)	37 (18.7)
BMI, kg/m^2	24.2 [21.5-26.8]	24.0 [22.0-26.6]	24.5 [21.7-27.5]
Heart rate, bpm	73 [65-83]	71 [65-81]	73 [65-82]
Systolic blood pressure, mmHg	127 [117-136]	124 [114-132]	125 [113-137]
O_2 saturation <90% (%)	1 (0.5)	1 (0.6)	15 (8.0)
NYHA class			
I (%)	193 (97.5)	189 (95.0)	152 (76.8)
II (%)	5 (2.5)	10 (5.0)	41 (20.7)
III (%)	0 (-)	0 (-)	5 (2.5)
IV (%)	0 (-)	0 (-)	0 (-)
Cardiac medication use	47 (23.7)	53 (26.6)	112 (56.6)
**Electrocardiography**			
Rhythm			
Sinus (%)	189 (95.5)	185 (93.0)	140 (70.7)
Paced (%)	5 (2.5)	11 (5.5)	28 (14.1)
Atrial fibrillation (%)	0 (-)	0 (-)	15 (7.6)
Other (%)	4 (2.0)	3 (1.5)	15 (7.6)
QRS-duration, msec	110 [98-126]	111 [99-144]	120 [101-151]
**Echocardiography**			
Left atrial volume, mL	38.6 [26.7-51.5]	38.3 [28.4-52.5]	52.5 [33.0-83.7]
LV end-diastolic volume, mL	116.8 [94.3-142.0]	111.3 [89.1-144.0]	119.5 [95.9-146.3]
LV end-systolic volume, ml	50.6 [38.8-61.7]	46.3 [37.7-61.7]	53.1 [38.6-73.3]
RA area, cm^2	19.3 [16.5-26.7]	20.0 [16.7-22.5]	27.2 [17.9-30.4]
RV end-diastolic area, cm^2	27.7 [23.4-33.9]	30.0 [23.0-39.6]	33.9 [25.4-40.2]
RV end-systolic area, cm^2	16.6 [12.1-19.8]	19.4 [12.2-25.8]	22.1 [14.5-30.2]
E/A ratio	1.5 [1.2-2.0]	1.5 [1.2-2.1]	1.5 [1.1-2.0]
E'wave, m/s	8.8 [7.1-10.2]	8.0 [6.9-9.6]	6.6 [5.4-8.6]
E/E' ratio	8.9 [7.5-12.0]	10.5 [8.4-14.4]	13.3 [8.9-18.0]
LV ejection fraction, %	58 [53-61]	57 [52-60]	53.8 [49.8-59.2]
Right ventricular fractional area change, %	41.0 ±9.3	39.9 ±10.8	33.8 ±12.2
Systemic ventricular function			
Normal (%)	138 (69.7)	99 (50.0)	94 (47.5)
Mildly impaired (%)	59 (29.8)	74 (37.4)	80 (40.4)
Moderately impaired (%)	1 (0.5)	21 (10.6)	18 (9.1)
Severely impaired (%)	0 (-)	4 (2.0)	6 (3.0)
Estimated RA-pressure			
5 mmHg (%)	134 (93.7)	129 (90.8)	107 (80.54
10 mmHg (%)	4 (2.8)	7 (4.9)	11 (8.3)
15 mmHg (%)	5 (3.5)	4 (2.8)	11 (8.3)
20 mmHg (%)	0 (-)	2 (1.4)	4 (3.0)
**Blood biomarkers**			
Hemoglobin, mmol/L	9.5 [8.9-9.9]	9.2 [8.5-9.7]	9.1 [8.4-9.6]

Hematocrit, L/L	0.45 [0.42-0.46]	0.44 [0.41-0.46]	0.43 [0.41-0.45]
MCV, fL	87 [85-89]	88 [86-90]	89 [86-92]
RDW, %	12.8 [12.4-13.3]	13.0 [12.6-13.4]	13.4 [12.8-14.1]
Urea, mmol/L	5.0 [4.3-5.9]	5.0 [4.2-5.9]	5.4 [4.4-6.4]
Creatinine, µmol/L	78 [70-86]	74 [65-83]	84 [66-83]
eGFR, ml/min/1.73m^2	90 [86-90]	90 [84-90]	87 [78-90]
Total cholesterol, mmol/L	4.6 [4.2-5.5]	4.7 [4.1-5.3]	4.7 [4.1-5.6]
LDL, mmol/L	2.9 [2.4-3.5]	2.8 [2.4-3.5]	2.9 [2.3-3.6]
HDL, mmol/L	1.3 [1.1-1.6]	1.4 [1.1-1.6]	1.4 [1.1-1.7]
NT-proBNP, pmol/L	5.0 [2.9-6.8]	15.2 [11.5-19.1]	84.6 [33.2-83.0]
hsTnT, ng/L	3.4 [2.1-5.2]	3.8 [2.1-6.2]	6.9 [3.8-11.4]
GDF-15, ng/L	543 [451-651]	604 [474-749]	812 [580-1326]
hsCRP, mg/L	0.9 [0.4-2.0]	1.3 [0.6-2.9]	2.5 [1.0-5.1]
ST-2, ng/mL	25.8 [19.1-33.7]	24.7 [18.1-31.0]	22.6 [16.4-31.6]
Galectin-3, ng/mL	12.1 [10.6-13.6]	12.1 [10.7-14.1]	13.8 [11.9-16.2]

Supplemental table 11: Congenital diagnosis per NT-proBNP tertile

Diagnosis	Tertile 1 (3.4-76.1 ngl/L, n=198)	Tertile 2 (80.0-208.9 ng/L, n=199)	Tertile 3 (213.1-6993.9 ng/L, n=198)
Tetralogy of Fallot	56 (28.3)	65 (32.7)	56 (28.3)
Aortic stenosis	62 (31.3)	47 (23.6)	28 (14.1)
Aortic coarctation	56 (28.3)	29 (14.6)	24 (12.1)
TGA-Mustard correction	2 (1.0)	24 (12.1)	39 (19.7)
Fontan circulation	5 (2.5)	13 (6.5)	18 (9.1)
TGA-Arterial switch	13 (6.6)	9 (4.5)	2 (1.0)
Congenitally corrected TGA	2 (1.0)	5 (2.5)	13 (6.6)
Rastelli/REV	1 (0.5)	6 (3.0)	4 (2.0)
Pulmonary arterial hypertension / Eisenmenger syndrome with underlying ASD	0 (-)	1 (0.5)	8 (4.0)
Functional univentricular heart	1 (0.5)	0 (-)	6 (3.0)

Supplemental figure 1: Kaplain Meier estimates of 10-year survival, heart-failure free survival, arrhythmia free survival and cardiovascular event free survival.

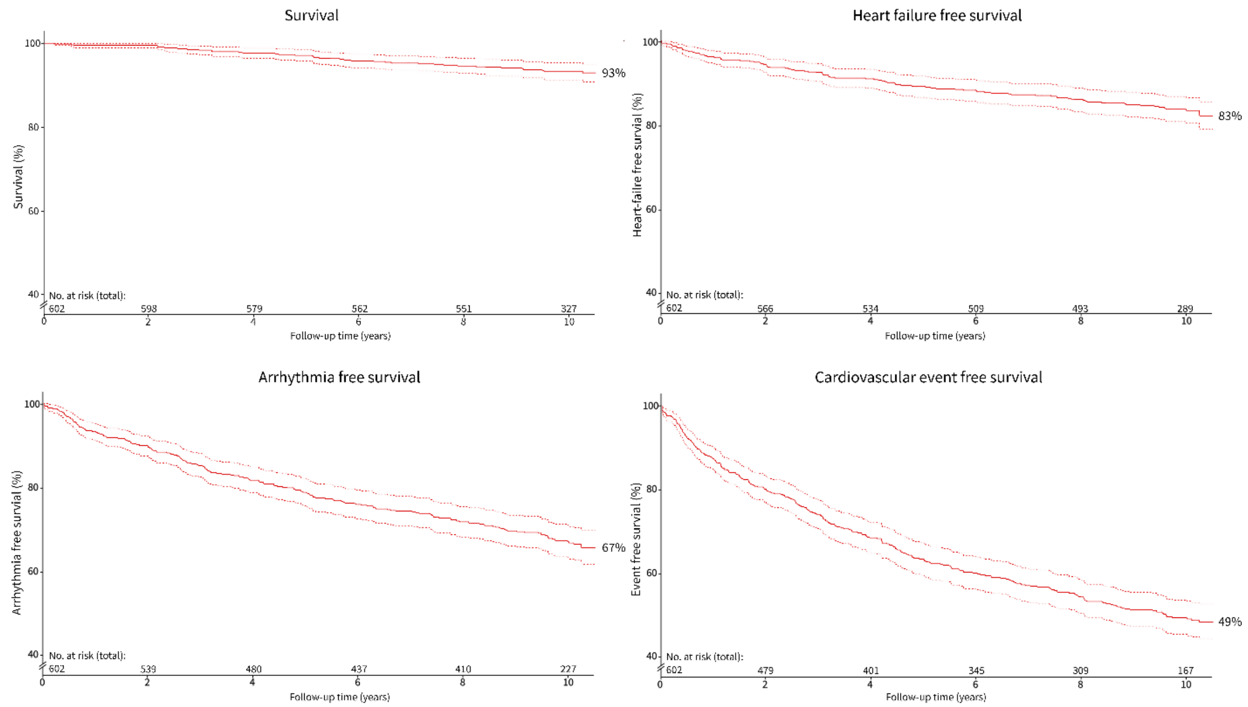

Supplemental figure 2: Survival and heart failure free survival per number of abnormal biomarkers. Included biomarkers: hematocrit, RDW, creatinine, total cholesterol, NT-proBNP, hsTnT, GDF-15, hsCRP, ST-2 and galectin-3

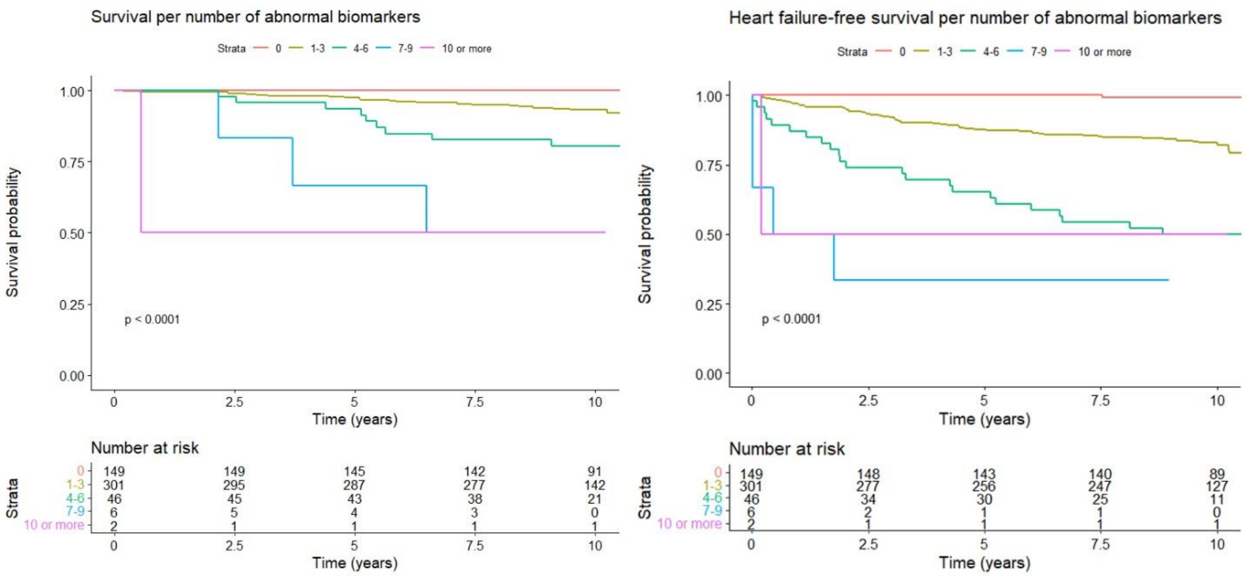

Supplemental figure 3: Correlations between measured blood biomarkers

	ST-2	Hemoglobin	Hematocrit	hs-Troponin T	Urea	Creatinine	MCV	HDL	Total Cholesterol	LDL	hs-CRP	Galectin-3	RDW	NT-proBNP	GDF-15
ST-2	1.00	0.33	0.35	0.22	0.15	0.21	0.00	−0.14	−0.10	−0.04	−0.06	0.01	0.02	−0.06	−0.04
Hemoglobin		1.00	0.94	0.16	0.16	0.34	0.08	−0.19	0.07	0.12	−0.19	−0.10	−0.18	−0.20	−0.13
Hematocrit			1.00	0.16	0.16	0.33	0.15	−0.20	0.05	0.10	−0.17	−0.08	−0.10	−0.14	−0.10
hs-Troponin T				1.00	0.31	0.29	0.03	−0.02	0.12	0.11	0.04	0.16	0.18	0.38	0.23
Urea					1.00	0.42	0.00	0.03	0.08	0.05	−0.05	0.06	0.06	0.11	0.11
Creatinine						1.00	−0.03	−0.13	0.01	0.02	−0.11	0.05	−0.03	−0.11	0.05
MCV							1.00	0.12	0.05	0.00	0.04	0.07	−0.06	0.23	0.22
HDL								1.00	0.18	−0.11	−0.16	−0.01	−0.09	0.04	−0.01
Total Cholesterol									1.00	0.90	0.09	0.12	0.08	0.03	0.07
LDL										1.00	0.10	0.10	0.08	−0.01	0.03
hs-CRP											1.00	0.22	0.22	0.28	0.21
Galectin-3												1.00	0.28	0.26	0.30
RDW													1.00	0.31	0.31
NT-proBNP														1.00	0.44
GDF-15															1.00
